# Assessment of dyspneic sensation in patients with type 2 diabetes

**DOI:** 10.3389/fendo.2023.1208020

**Published:** 2023-08-10

**Authors:** Chadia Mizab, Enric Sánchez, Liliana Gutiérrez-Carrasquilla, Núria Balsells, Anaïs Arqué, Raquel Ruano, Magda Mateu, Marta Zorzano-Martínez, Anna Pomés, Esther García-Aguilera, Raquel Martí, José María Manzanares, Cristina Hernández, Rafael Simó, Albert Lecube

**Affiliations:** ^1^ Endocrinology and Nutrition Department, University Hospital Sant Joan de Reus, Reus, Spain; ^2^ Endocrinology and Nutrition Department, University Hospital Arnau de Vilanova, Lleida, Spain; ^3^ Obesity, Diabetes and Metabolism (ODIM) Research Group, Institut de Recerca Biomèdica de Lleida (IRBLleida), Lleida, Spain; ^4^ Medicine and Surgery Department, University of Lleida, Lleida, Spain; ^5^ Endocrinology and Nutrition Department, Hospital Universitari Vall d’Hebron, Barcelona, Spain; ^6^ Diabetes and Metabolism Research Unit, Vall d’Hebron Institut de Recerca (VHIR), Barcelona, Spain; ^7^ Centro de Investigación Biomédica en Red de Diabetes y Enfermedades Metabólicas Asociadas (CIBERDEM), Instituto de Salud Carlos III (ISCIII), Madrid, Spain

**Keywords:** diabetes complications, dyspneic sensation, type 2 diabetes, respiratory symptoms, questionnaires

## Abstract

**Introduction:**

Individuals with type 2 diabetes (T2D) should be considered a susceptible group for pulmonary dysfunction. So, we aimed to evaluate the sensation of breathlessness in this population by administering two well-validated questionnaires.

**Methods:**

This is a crosssectional study with 592 people without known respiratory disease (353 with T2D) who answered the modified Medical Research Council (mMRC) questionnaire. In addition, 47% also responded to the St George Respiratory Questionnaire, a specific instrument designed to be applied to patients with obstructive airway disease.

**Results:**

Patients with T2D showed a higher mMRC score in comparison to the control group [1.0 (0.0 – 4.0) vs. 0.0 (0.0 – 4.0), p<0.001]. A higher prevalence of subjects with mMRC ≥2 was observed in T2D that in the control group (20.2% *vs*. 11.6%, p=0.004). Participants with T2D and mMRC ≥2 showed a higher HbA1c (8.2 ± 1.6% vs. 7.8 ± 1.6%, p=0.048), longer T2D evolution and higher prevalence of nephropathy. In the multivariate analysis, the presence of T2D [OR=1.95 (1.19 to 3.22), p=0.008] in all the population, and HbA1c [OR=1.19 (1.01 to 1.41), p=0.034] and the presence of diabetic nephropathy [OR=2.00 (1.14 to 3.52), p=0.015] in patients with T2D, predicted a mMRC ≥2. Finally, no differences were observed regarding the SGRQ score among groups.

**Conclusions:**

Patients with T2D showed a greater sensation of dyspnea than subjects with normal carbohydrate metabolism. Risk factors included poor metabolic control and the presence of renal disease.

## Introduction

Over the last decade, ample evidence has accumulated suggesting that type 2 diabetes (T2D) has a negative impact on lung function and nocturnal breathing (1, 2). Thus, different population-based studies have recurrently reported a decrease in spirometric results in patients with T2D, which is between 8 and 10% of the theoretical values (2, 3). A decrease in forced vital capacity, forced expiratory volume in first second, and diffusing capacity of the lungs for carbon monoxide have been described (4). Likewise, it has been described how pulmonary function impairment is present even in the prediabetes state (5). It has also been shown that the deterioration of lung function is associated with the degree of metabolic control (4, 6). In this way, optimization of metabolic control, independent of weight loss, has been associated with an improvement in spirometric values (7). In addition, data from the *Fremantle Diabetes Study* also showed that the duration of T2D was associated with reduced forced expiratory volume in first second and peak expiratory flow (8).

In the other hand, when the sleep period is evaluated, T2D also appears as an independent risk factor for experiencing greater hypoxemia, with a greater percentage of the sleep period with oxygen saturations below 90%, a greater number of episodes of desaturation and even a greater number of micro awakenings (4, 9). The deleterious impact of T2D on sleep breathing has also been revealed in the prediabetes stage (10). In fact, questionnaires as the Pittsburgh Sleep Quality Index (PSQI) and the Epworth Sleepiness Scale (ESS) have revealed how the presence of T2D adversely affect sleep quality and are independent risk factors for daytime sleepiness (11).

However, there are few data on the impact of altered spirometric parameters on the sensation of breathlessness in patients with T2D, and whether it is associated with the other late complications of diabetes. In addition, these studies have been conducted using different tools and including patients with pulmonary disease or cardiac disease (12, 13). For this reason, our aim was to evaluate the clinical impact of T2D on dyspneic sensation by administering two well-validated questionnaires as the modified medical research council (mMRC) dyspnea scale and the Saint George’s Respiratory Questionnaire (SGRQ) (14, 15). In addition, the coexistence of other classical chronic complications of T2D has also been evaluated.

## Material and methods

### Statement on ethics

The human ethics committee from the Arnau de Vilanova University Hospital and the Sant Joan de Reus University Hospital (CEIC-2173 and CEIM173/2019, respectively) approved the study. Written informed consent was obtained from all participants and the study was conducted according to the ethical guidelines of the Helsinki Declaration.

### Design of the study and description of the study population

**Figure 1:**
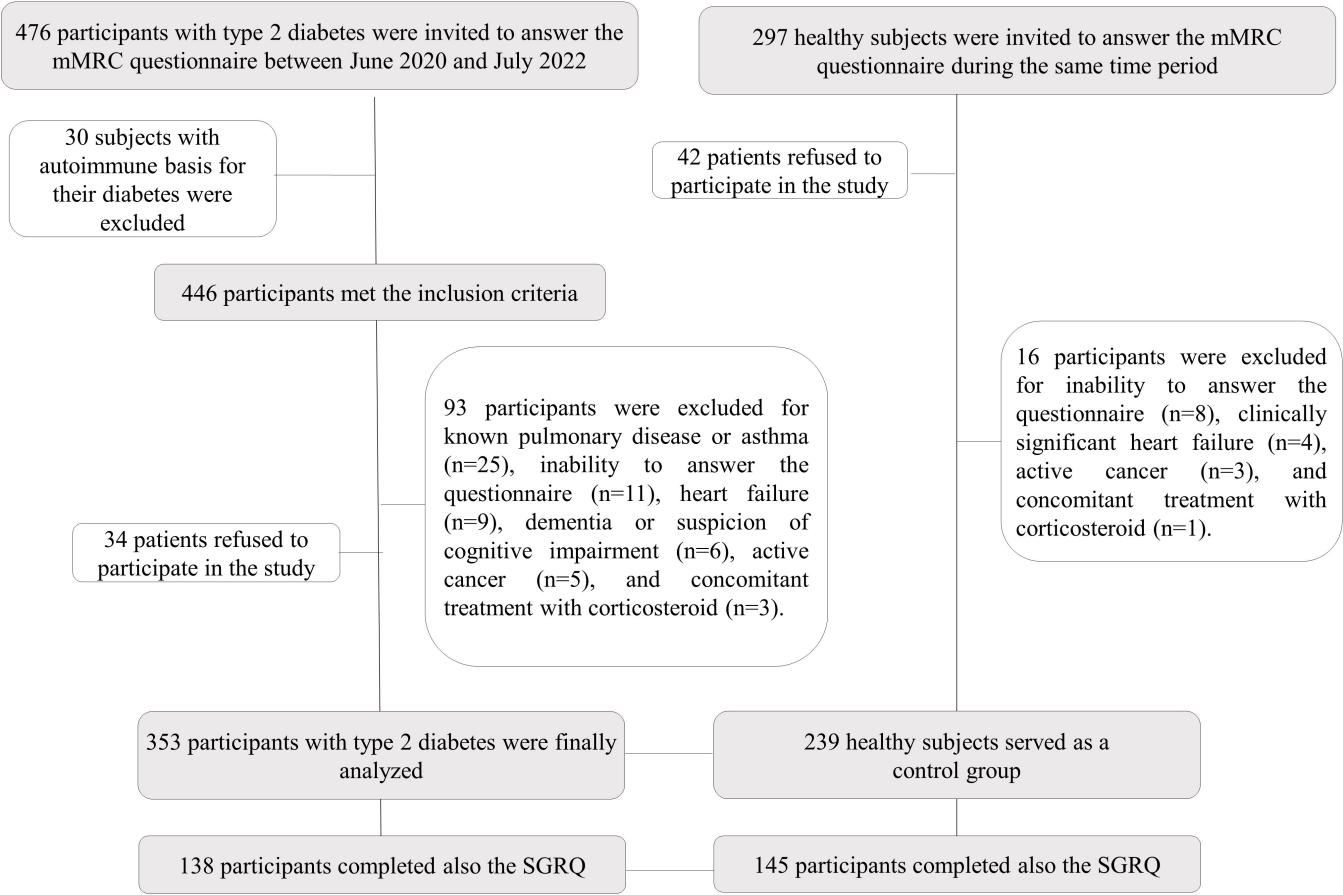
Flow Diagram for the study population.

This is a crosssectional study to assess the dyspnea sensation in patients with T2D. Therefore, 476 patients were invited to participate from both University Hospitals from June 2020 to July 2022. The inclusion criteria were: (i) men and women, (ii) type 2 diabetes with at least three years of known disease, (iii) age between 45 and 75 years old, and (iv) available clinical and analytical data. Among the 446 participants who met the inclusion criteria, we excluded 93 for the following reasons: known chronic pulmonary disease or asthma (n=25), inability to answer the questionnaire correctly (n=11), clinically significant heart failure (n=9), dementia or suspicion of cognitive impairment (n=6), active cancer (n=5), and concomitant treatment with corticosteroid (n=3). In addition, 34 patients refused to participate in the study. No pregnant women were evaluated (**Figure 1**). In addition, data from 239 healthy subjects with normal carbohydrate metabolism and without pulmonary disease were recruited among the employees of our institutions and relatives of patients with T2D. We tried to match these individuals with patients with T2D by age (within 3 years range) and BMI (within 2.0 kg/m range). However, it was not possible to match both populations by sex. The main clinical features of the entire population are displayed in **Table 1**.

**Table 1:** Main clinical characteristics and comorbidities of the study population responding to the modified medical research council (mMRC) dyspnea scale and the Saint George’s Respiratory Questionnaire (SGRQ) according to the presence of type 2 diabetes.

	mMRC			SGRQ			
	T2D	Control	p	T2D	Control	p	
n	353	239		138	145		
Women, n (%)	167 (47.4)	160 (67.2)	<0.001	73 (45.3)	98 (64.9)	0.001	
Age (years)	61.8 ± 11.7	60.2 ± 10.4	0.068	61.8 ± 11.3	60.3 ± 9.9	0.217	
Active smoker, n (%)	56 (15.9)	35 (14.7)	0.707	15 (10.8)	19 (12.9)	0.593	
BMI (kg/m^2^	31.4 ± 6.0	31.0 ± 8.0	0.545	38.6 ± 8.2	38.0 ± 9.0	0.531	
Body fat mass (%)	38.8 ± 8.4	38.8 ± 8.6	0.910	23 (14.2)	20 (13.2)	0.790	
FPG (mg/dl)	162.2 ± 65.4	96.5 ± 15.0	<0.001	149.0 ± 64.1	96.0 ± 15.3	<0.001	
HbA1c (%)	7.9 ± 1.6	5.6 ± 0.4	<0.001	7.6 ± 1.5	5.6 ± 0.3	<0.001	
Diabetes evolution (years)	14.9 ± 10.6			14.6 ± 10.9			
Diabetic retinopathy, n (%)	81 (22.9)			53 (32.9)			
Diabetic nephropathy, n (%)	124 (35.2)			35 (21.7)			
Macroangiopathy, n (%)	28 (7.9)			11 (6.8)			

BMI, body mass index; FPG, fasting plasma glucose.

Diagnosis of diabetes was done as stated by the American Diabetes Association guidelines (16). The study variables were obtained from the computerized clinical history of both centers: sex, age, anthropometry, and analytical parameters (glucose, HbA1c, glomerular filtration rate and albuminuria). Smoking habit (smokers who stopped smoking ≥1 year prior to recruitment were considered former smokers) was also assessed. Diabetic retinopathy was diagnosed whether a fundus examination done by indirect ophthalmoscopy or fundus photographs within at least two years before inclusion documented vascular abnormalities. Kidney disease was diagnosed according to KDIGO guidelines (17). No data were available on the presence of neuropathy. Finally, we used the Clínica Universidad de Navarra-Body Adiposity Estimator (CUN-BAE) to calculate the percentage of total body fat (18).

### Questionnaires to evaluate dyspneic sensation

The modified medical research council (mMRC) dyspnea Scale is a questionnaire that assess perceived breathlessness on daily activities (14). The mMRC is a 5-point (0–4) scale based on the severity of dyspnea that ranges from grade 0 (“I only get breathless with strenuous exercise”) to grade 4 (“I am too breathless to leave the house or I am breathless when dressing”). Intermediate grades include “I get short of breath when hurrying on level ground or walking up a slight hill” (grade 1), “On level ground, I walk slower than people of the same age because of breathlessness, or I have to stop for breath when walking at my own pace on the level” (grade 2), and “I stop for breath after walking about 100 yards or after a few minutes on level ground” (grade 3). A total score of three or more can be used to predict hospitalization and exacerbation in patients with chronic obstructive pulmonary disease (19). In our population of patients with T2D without known pulmonary diseases we used the grade 2 as a cutoff point to diagnose positive dyspnea sensation (20). Moreover, the SGRQ is a standardized selfcompleted questionnaire for measuring impaired health and perceived wellbeing in respiratory tract diseases (15). It has 50 items with 76 weighted responses. Total score ranges from 0 to 100, with higher results indicating more respiratory limitations. This questionnaire also assesses dyspnea in 3 dimensions: (i) frequency and severity of respiratory symptoms (symptoms), (ii) limitation of activity owing to the symptoms (activity), and (iii) psychological and social functioning modifications (impact). The mMRC, the simplest questionnaire, was administered first, followed by the SGRQ to all participants. As described, the SGRQ is a longer and more complex questionnaire, which finally only 47.8% of the study population was able to complete correctly (**Table 1**).

### Statistical analysis

A normal distribution of the variables was verified *via* the Kolmogorov–Smirnov test. Consequently, data were expressed as the mean ± SD, median (range) or as percentages. The major clinical data across the two groups were compared using Student’s t-test for continuous variables, and the Mann-Whitney U test for skewed data. Chi-squared test was done for categorical variables. Moreover, the link between variables was evaluated using the Pearson correlation test or ANOVA. A multivariable logistic regression model for the presence of a positive dyspnea sensation (mMRC ≥2) was done both in the entire population (including age, sex, BMI, smoking habit and the presence of T2D as confounding variables) and in patients with T2D (including known evolution of T2D, sex, BMI, smoking habit, HbA1c and the presence of diabetic nephropathy as confounding variables). Calibration of model was assessed using the Hosmer–Lemeshow test of fit and the area under the Receiver Operating Characteristic (ROC) curve. All *p* values were based on a 2-sided test for statistical significance. Significance was accepted as a *p* value less than 0.05. Statistical analyses were performed using the SPSS software (IBM SPSS Statistics for Windows, Version 27, Armonk, NY, USA).

## Results

**Figure 2:**
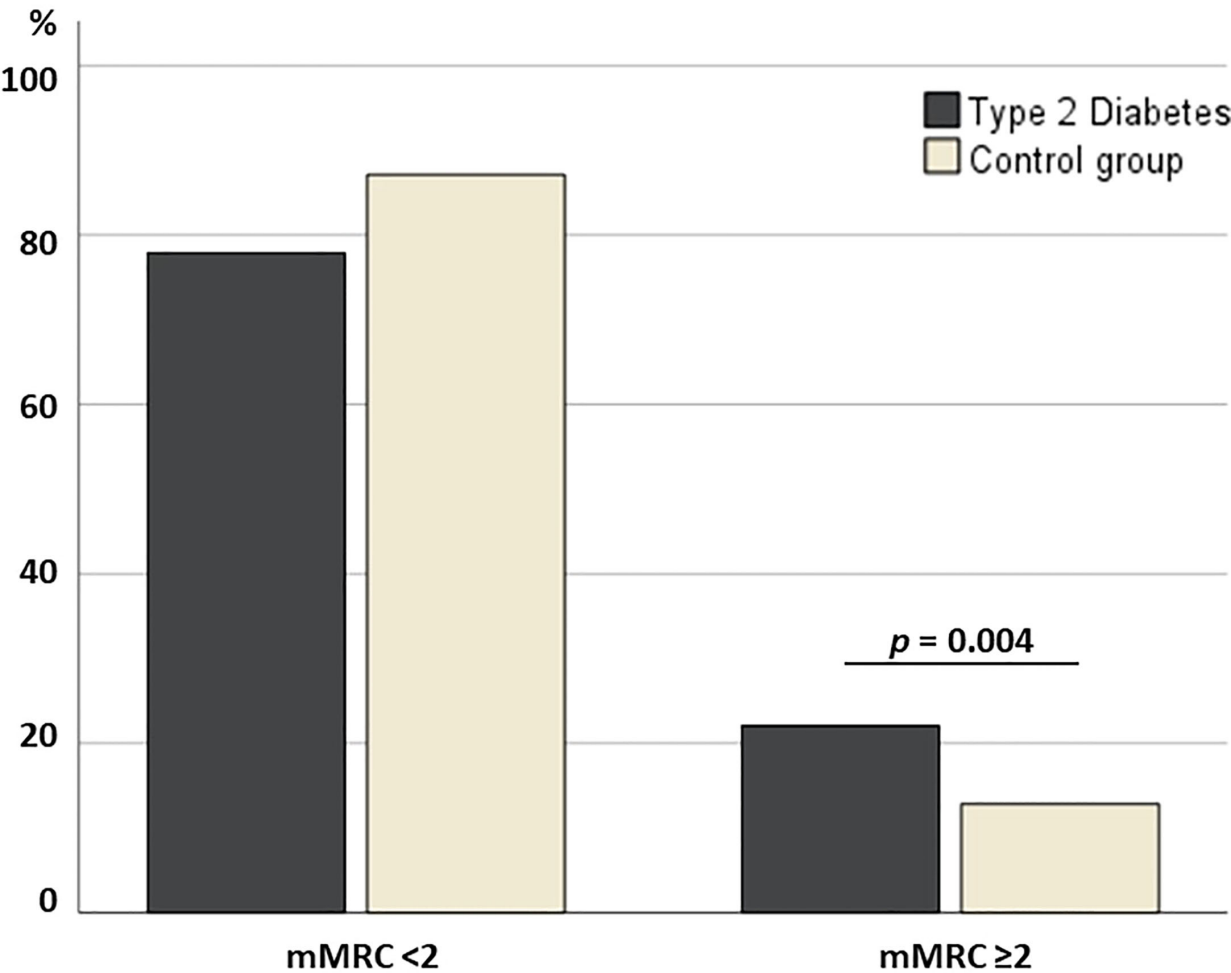
Bar plot displaying the prevalence of subjects with dyspneic sensation.

Patients with T2D showed a statistically higher mMRC score in comparison to the control group [1.0 (0.0 – 4.0) vs. 0.0 (0.0 – 4.0), p<0.001] (**Table 2**). Additionally, a higher prevalence of subjects with mMRC ≥2 was observed in patients with T2D than in the control group (20.2% vs. 11.6%, p=0.004) (**Figure 2**). When only participants with T2D were assessed, those with mMRC ≥2 were mainly women, older and heavier, showed a higher HbA1c (8.2 ± 1.6% vs. 7.8 ± 1.6%, p=0.048) without differences in FPG, more years with diabetes and higher prevalence of nephropathy (**Table 3**).

**Table 2:** Results of Modified medical research council questionnaire according to the presence of type 2 diabetes.

	Type 2 diabetes	Control	p
mMRC score	1.0 (0.0 – 4.0)	0.0 (0.0 – 4.0)	<0.001
Grade 0, n (%)	175 (49.5)	155 (64.8)	
Grade 1, n (%)	100 (28.3)	53 (22.1)	
Grade 2, n (%)	43 (12.1)	15 (6.2)	
Grade 3, n (%)	31 (8.7)	14 (5.8)	
Grade 4, n (%)	4 (1.1)	2 (0.8)	0.004 *

mMRC, Modified medical research council questionnaire; * ANOVA

**Table 3:** Clinical characteristics of patients with type 2 diabetes according to the presence of a positive dyspneic sensation defined by a modified Medical Research Council questionnaire ≥2.

	mMRC ≥2	mMRC <2	p
n	78	275	
Women, n (%)	50 (64.1)	117 (42,5)	0.001
Age (years)	66.3 ± 10.7	60.5 ± 11.7	<0.001
BMI (kg/m^2^)	33.0 ± 7.1	30.9 ± 5.6	0.019
Body fat mass (%)	42.6 ± 8.4	37.6 ± 8.1	<0.001
Active smoker, n (%)	15 (19.2)	41 (14.9)	0.363
FPG (mg/dl)	168.9 ± 69.9	159.5 ± 63.9	0.301
HbA1c (%)	8.2 ± 1.6	7.8 ± 1.6	0.048
Diabetes evolution (years)	17.5 ± 11.6	14.1 ± 10.2	0.012
Diabetic retinopathy, n (%)	22 (28.2)	59 (21.4)	0.281
Diabetic nephropathy, n (%)	38 (48.7)	86 (31.2)	0.009
Macroangiopathy, n (%)	8 (10.2)	20 (7.2)	0.635

mMRC, modified Medical Research Council questionnaire; BMI, body mass index; FPG, fasting plasma glucose.

**Figure 3:**
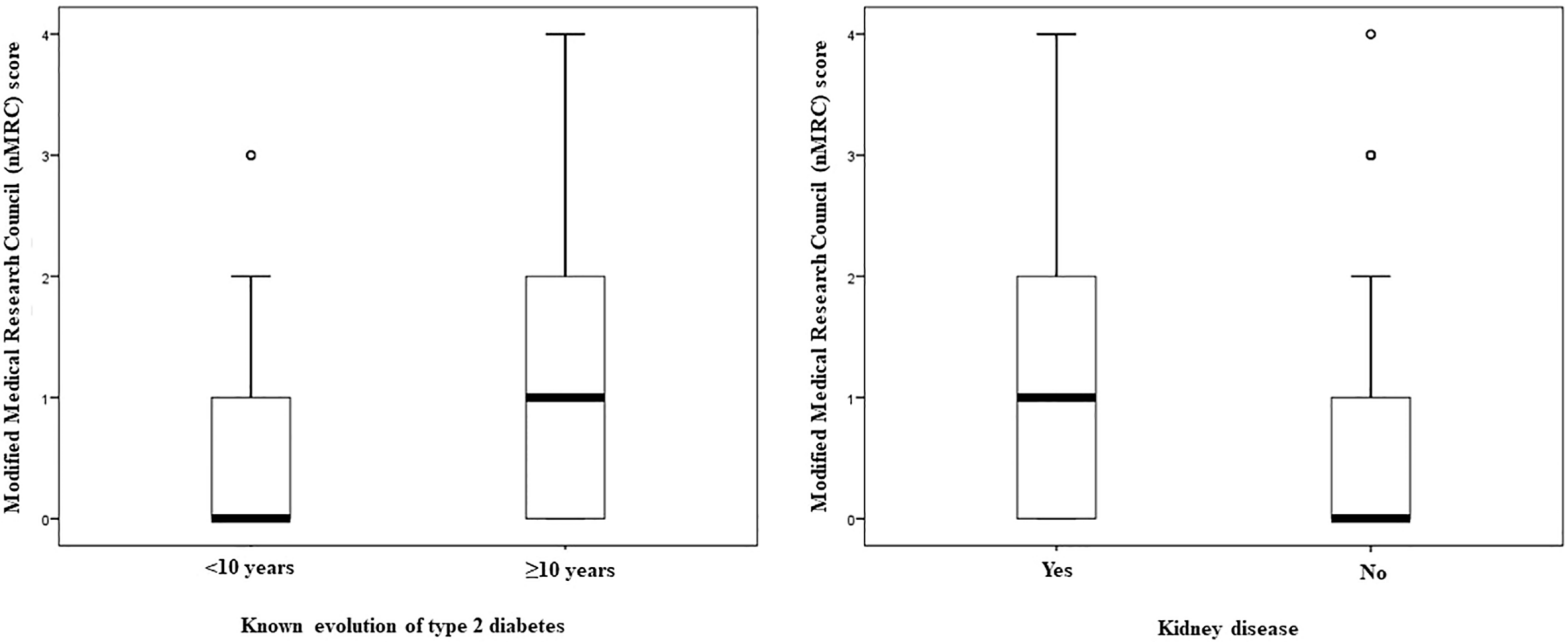
Box plots displaying the modified Medical Research Council score of subjects with type 2 diabetes according to years after diabetes diagnosis and the presence of kidney disease.

When the known evolution of T2D was less than 10 years, the prevalence of mMRC ≥2 was 14.1%, increasing to 25.5% when the evolution was ≥10 years (p=0.018) (**Figure 3**). In addition, the mMRC score was significantly higher in those patients with T2D and more than 10 years of evolution than in those with less time of disease [1.0 (0.0 – 4.0) vs. 0.0 (0.0 – 3.0), p=0.043]. Likewise, the prevalence of mMRC ≥2 increased from 18.3% in patients without kidney disease to 30.6% in those with diabetic nephropathy (p=0.009). Similarly, in patients with T2D the mMRC score raised from 0.0 (0.0 – 4.0) in those without to 1.0 (0.0 – 4.0) in those with nephropathy (p=0.047) (**Figure 3**). However, no differences were observed between patients with or without retinopathy (p=0.081) or macrovascular disease (0.467) in the mMRC score. In the multivariate analysis, when all the participants in the study were included, age, sex, BMI, active smoking habit and the presence of T2D [OR=1.95 (1.19 to 3.22), p=0.008] predicted the presence of a mMRC ≥2 (**Table 4**). When the analysis was restricted to patients with T2D, HbA1c [OR=1.19 (1.01 to 1.41), p=0.034] and the presence of diabetic nephropathy [OR=2.00 (1.14 to 3.52), p=0.015], but not years of evolution of the disease, appeared to be independently related with a mMRC ≥2 (**Table 4**).

**Table 4:** A multinomial logistic regression model for presence of a modified Medical Research Council ≥2 in both the entire population and restricted to patients with type 2 diabetes.

mMRC ≥ 2		Entire population	
		OR (95% CI)	p value
Age (years)		1.05 (1.03 to 1.08)	<0.001
Sex Women		Reference	
Men		1.99 (1.24 to 3.18)	0.004
Body mass index (kg/m^2^)		1.08 (1.05 to 1.12)	<0.001
Smoking habit Never		Reference	
Current or former		1.89 (1.03 to 3.46)	0.038
Type 2 diabetes No		
Yes		1.95 (1.19 to 3.22)	0.008
Pearson's Goodness-of-Fit Test			0.452
Area under the ROC curve		0.72 (0.67 to 0.77)	<0.001

mMRC ≥ 2		Patients with type 2 diabetes	
		OR (95% CI)	p value
Evolution of T2D (years)		1.024 (0.998 to 1.05)	0.073
Sex Women		Reference	
Men		2.48 (1.41 to 4.35)	0.002
BMI (kg/m^2^)		1.06 (1.01 to 1.10)	0.009
Smoking habit Never		Reference	
Current or former		1.93 (0.94 to 3.96)	0.072
HbA1c (%)		1.19 (1.01 to 1.41)	0.034
Diabetic nephropathy No		Reference	
Yes		2.00 (1.14 to 3.52)	0.015
Pearson's Goodness-of-Fit Test			0.489
Area under the ROC curve		0.75 (0.65 to 0.86)	<0.001

T2D, type 2 diabetes; BMI, body mass index; HbA1c, glycated hemoglobin.

Gray shading in Type 2 diabetes line differetiates the two multivariate analysis performed in the study.

Regarding the SGRQ, no differences were observed in the total score among groups [14.3 (0.0 - 75.3) in T2D vs. 12.2 (0.0 - 65.6) in controls, p=0.923] nor in the symptoms [8.8 (0.0 - 61.7) vs. 8.8 (0.0 - 75.7), p=0.698], activity [23.8 (0.0 - 100.0) vs. 18.4 (0.0 - 100.0), p=0.690] and impact [4.6 (0.0 - 69.5) vs. 4.8 (0.0 to 57.2), p=0.827] domains of the questionnaire. In the univariate analysis, the total score of the SGRQ showed a significant correlation with BMI (r=0.212, p<0.001) and total body fat (r=0.214, p<0.001), but not with age (r=0.054, p=0.327), FPG (r=0.056, p=0.396) or HbA1c (r=0.021, p=0.794).

## Discussion

This is a cross-sectional study that provides evidence in favor of the idea that T2D has a negative impact on lung function that is associated with an increased selfperceived sense of dyspnea. This sensation would appear during activities of daily living and can be assessed with simple questionnaires.

Even though previous studies have shown that patients with T2D have decreased lung capacity compared to the general population, few have so far evaluated the clinical impact that diabetes may have on the sensation of dyspnea (21). It has been suggested that since the pulmonary microvascular bed is the largest in humans, great involvement is required for any type of symptom and disability to appear (4). Thus, the involvement of other organs and systems with a smaller microvasculature, such as the retina or the kidney, would appear more quickly in patients with T2D, despite a similar severity of anatomic involvement (20). Therefore, the deleterious impact of diabetes on the lungs would result in dysfunctions that would remain undiagnosed for a long period of time if screening is not proposed in routine clinical practice (4).

Dyspnea is a heterogeneous symptom that can only be known through a patient’s report and can be present quantitatively and qualitatively different among patients (22). However, knowledge of its evolution is important for the diagnosis and monitoring of patients with T2D who will require a complementary study of their pulmonary function. In this way, the mMRC scale is a simple and quick self-assessment tool that measure the degree of disability that dyspnea poses to daily activities with a simple question (14). Kopf et al. showed a progressive increase in the mMRC score from controls (n=48) to patients with prediabetes (n=68), patients with newly diagnosed T2D (n=29) and patients with long term diabetes (n=110) (23). This result is in line with the fact than in the entire population of our study the presence of T2D appeared to be an independent factor to score ≥2 in the mMRC. In parallel, the 14.1% to 25.5% increase that we have described when the known duration of T2D exceeds 10 years support that the symptomatic dyspnea appears progressively in patients with T2D, in whom disease evolution must be added to other factors such as sex and BMI.

In Italy, in the framework of the GEIRD (*Gene Environment Interactions in Respiratory Diseases*) study, De Santi et al. showed how a group of 259 patients with T2D reported more frequently than the general population a dyspnea that limited walking on level ground (grade 2 mMRC dyspnea) (12). In fact, the odds ratio of grade 2 mMRC dyspnea among patients with T2D was almost four [3.92 (3.28 - 4.68), p <0.001] in the 4554 age group and decreased to 1.79 (1.68 - 1.91; p <0.001) in the 75 - 84 age group. More recently, the *Canadian Longitudinal Study on Aging* evaluated the potential risk factors for dyspnea (measured by mMRC) in 28,854 adults aged 45 to 85 years old (21). Even the population also comprised subjects with lung disease, depression and heart disease, the study described how the adjusted odds ratio for dyspnea “walking on flat surfaces” was highest in patients with T2D (21). It is important to note that both studies included participants with asthma and chronic obstructive pulmonary disease, so the origin of dyspnea cannot be attributed exclusively to the presence of diabetes. To avoid this bias, we were careful to exclude other causes that could favor the presence of dyspnea, such as pulmonary disease or clinically significant heart failure. However, we could not rule out the presence of heart failure with preserved ejection fraction that could also favor respiratory symptomatology (24).

The Hispanic Community Health Study/Study of Latinos (HCHS/SOL) is a large, multicenter observational study that included 2,300 T2D patients without lung disease and assessed symptomatic lung impairment using the Baseline Dyspnea Index (BDI) questionnaire, which provides measures significantly related to the mMRC (25, 26). Key results showed a significantly higher breathlessness score in people with T2D compared to the population without diabetes [0.60 (0.49–0.71) vs. 0.41 (0.34–0.49), p<0.001]. This study was the first to show that the impairment in the dyspnea score correlated with the ratio, rising by 0.02 ± 0.006 per 100 mg/g increase in those patients without previous disease (25). In this way, Vracko et al. described in the year 1979 how the thickness of the epithelial and capillary basal laminae of alveoli correlated significantly with thickness of basal laminae in renal tubules (27). In our study, the prevalence of mMRC ≥2 doubled from 18.3% to 30.6% in patients with T2D with and without diabetic kidney disease. These data were corroborated by multivariate analysis, in which the presence of diabetic nephropathy significantly increased the risk of obtaining a mMRC score equal or higher than 2.

None of the studies cited above have established a correlation between metabolic control and dyspnea symptoms in patients with T2D (12, 21). However, in our cohort of patients with T2D, not only did those with an mMRC score ≥2 have significantly higher HbA1c values than those with a lower mMRC score, but HbA1c was also an independent factor related to the presence of positive dyspnea sensation in the multivariate analysis. Taken together, our results reinforce the negative impact of T2D on lung function and the importance of achieving good metabolic control in the treatment of this complex disease.

Finally, when the Saint George’s respiratory questionnaire was administered to participants with and without T2D, no differences were found between groups not only in the total score, but also in the dimensions of symptoms, activity and impact. These contradictory results could be partly explained by the fact that this questionnaire is a specific instrument designed to be applied to patients with obstructive airway disease, capable of measuring the impact on patients’ general health, daily life and perceived wellbeing. Therefore, when administered to patients without known lung disease, this instrument may not be sensitive and specific enough to detect small differences between patients with and without T2D.

This study has some limitations that need to be considered. The first is that dyspnea is a subjective sensation that changes according to each patient’s perception of their state of health. However, it is important to know how patients with diabetes perceive and self-report this sensation, as it can help us to identify those who need a more extensive evaluation of their lung function. In addition, the lack of information on the presence of established neuropathy in our cohort of patients with type 2 diabetes prevents us from ruling out that low-grade neuropathy translates into a feeling of increased dyspnea. Secondly, we have not correlated the sensation of dyspnea with lung function, so we cannot state that patients with the highest score on the mMRC are those who also have the most affected spirometric values. However, previous studies have well established that the mMRC scale is recommended for assessing dyspnea and an mMRC scale grade equal to or greater than 3 can be used to predict hospitalization and exacerbation in patients with chronic obstructive pulmonary disease (14, 19). Third, the treatment of T2D has not been evaluated. In this regard, there is evidence that glucagon-like peptide 1 receptor agonists, such as liraglutide, have a positive effect on lung function, independent of the weight reduction associated with their use (28). Finally, it should be remembered that our population of patients with diabetes was recruited from the outpatient clinics of two university hospitals, being heterogeneous in terms of factors such as sex and age, so it is possible that the results obtained cannot be generalized to other groups of patients.

## Conclusions

Patients with T2D showed a greater sensation of dyspnea than subjects with normal carbohydrate metabolism. Risk factors included male sex and higher BMI, but also poor metabolic control and the presence of renal disease. These data support the fact that the lungs should be considered a target organ for late complications of T2D and that, consequently, the symptomatology that may result from pulmonary dysfunction should be investigated.

## Data availability statement

The original contributions presented in the study are included in the article/supplementary material. Further inquiries can be directed to the corresponding author.

## Ethics statement

The studies involving human participants were reviewed and approved by The human ethics committee from the Arnau de Vilanova Hospital University and the Sant Joan de Reus University Hospital (CEIC2173 and CEIM173/2019, respectively). The patients/participants provided their written informed consent to participate in this study.

## Author contributions

CM and ES contributed equally to this work. All authors contributed to the article and approved the submitted version.
